# Seasonally structured drone data for shoreline change around a hybrid living shoreline project

**DOI:** 10.1038/s41597-025-06310-z

**Published:** 2025-12-12

**Authors:** Sarah Pettyjohn, Hannah Sirianni, Matthew J. Sirianni, Brendan M. J. Burchi, Rachel K. Gittman

**Affiliations:** 1 Department of Earth, Environment and Planning, East Carolina University, Greenville, NC 27858 USA; 2 Department of Biology, East Carolina University, Greenville, NC 27858 USA; 3 Coastal Studies Institute, Wanchese, NC 27954 USA

**Keywords:** Environmental impact, Natural hazards, Geomorphology

## Abstract

Nature-based shoreline protection projects are becoming increasingly common, but many monitoring programs capture only infrequent, post-construction snapshots that overlook seasonal dynamics. This dataset provides a structured, high-frequency record (2023–2025) of shoreline change at Sugarloaf Island, North Carolina, based on 16 drone surveys conducted before, during, and after the installation of Wave Attenuation Devices (WADs) and oyster breakwaters. Each seasonal interval includes paired east and west island surveys, RTK- GNSS ground control, and digitized stabilization-structure locations. Collected imagery was processed using a Structure-from-Motion and Multi-View Stereo photogrammetric workflow to produce dense point clouds (>700 pts/m²), 0.05 m resolution digital elevation models, and 0.007 m orthomosaics. Spatial accuracy, evaluated through 100-run Monte Carlo simulations, yielded horizontal RMSE = 0.008 to 0.044 m and vertical RMSE = 0.03 to 0.089 m across all surveys. This dataset establishes a seasonally structured, high-accuracy drone record of hybrid living shoreline evolution that supports shoreline stabilization and coastal resilience research.

## Background & Summary

Estuarine barrier islands provide essential ecological and geomorphic functions in semi-enclosed coastal systems. These islands buffer mainland shorelines and support intertidal habitats while modulating local hydrodynamics. Compared to ocean-facing island shorelines, estuarine shorelines lack the protective dune elevation found along more exposed coasts. These traits increase their susceptibility to land loss through erosion, overwash, and channel migration during prolonged storm events^1,2^.

Despite their importance, estuarine barrier islands are often omitted from national shoreline change inventories, particularly in low-energy systems with narrow or discontinuous geomorphic features^3^. Subtle elevation gradients can be difficult to detect in moderate-resolution DEMs, which are less sensitive to gentle slopes. Foundational products such as the USGS National Elevation Dataset^4^ and more recent 3DEP LiDAR-derived datasets have significantly improved topographic coverage in many coastal areas. For example, 1-m LiDAR data from 2016 and 2020 were used to document estuarine shoreline erosion in North Carolina^5^. However, the multi-year acquisition frequency of these datasets limits their use for detecting short-term or seasonal morphological change. This constraint is especially significant in low-relief estuarine environments, where subtle transitions between marsh, flat, and intertidal zones require both high spatial resolution and frequent repeat surveys. Regional classification datasets^6^ provide important baselines for shoreline type and habitat, yet they do not capture seasonal-scale morphological dynamics that influence stabilization outcomes.

Drone-based Structure-from-Motion (SfM) photogrammetry, coupled with Multi-View Stereo (MVS) surface reconstruction, provides a practical solution, enabling affordable, on-demand, and repeatable surveys with sub-decimeter horizontal and vertical accuracy. This combination of high-definition mapping and flexible revisit intervals surpasses the capabilities of satellite- or airborne-based remote sensing platforms, making it well-suited for capturing seasonal geomorphic and ecological changes. We build on SfM-MVS approaches widely applied across diverse coastal environments to reconstruct landform morphology and detect fine-scale elevation and habitat change^7,8^.

**Fig. 1:**
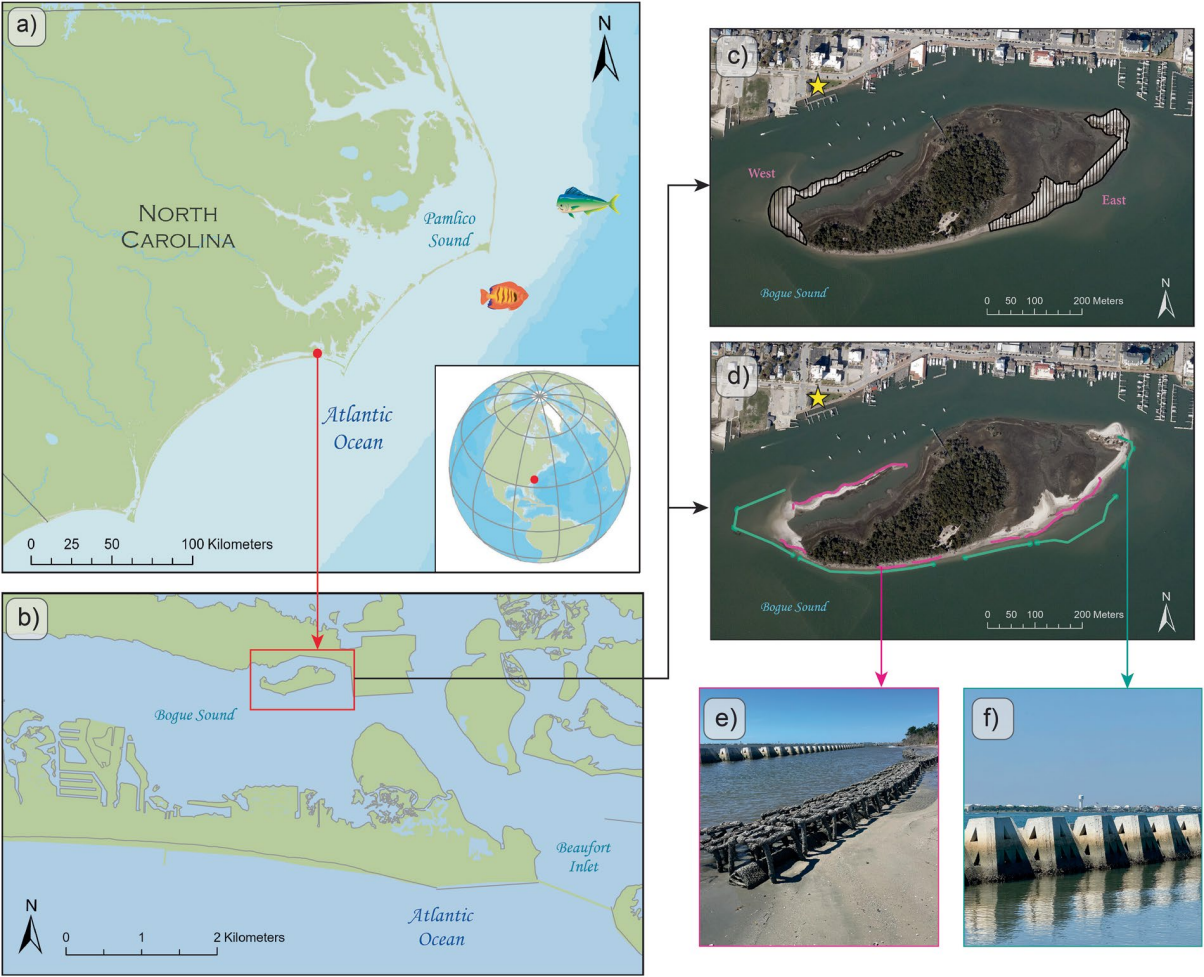
Map of North Carolina coastal region at different scales. (**a**) The red dot indicates where Sugarloaf Island is located, (**b**) location of Sugarloaf Island in Bogue Sound, (**c**) RGB imagery of the island from 2020 (https://coast.noaa.gov/dataviewer/#/; accessed on 5 May 2024) with flight plans representing the area surveyed as well as a yellow star indicating the location of Jaycee Park where repeat control points were collected, (**d**) locations of the ~1000 m of WADs (sea green) and ~740 m of oyster reefs (magenta) around the island, and ground photos of the (**e**) oyster reefs and (**f**) WADs.

These SfM-MVS techniques are particularly valuable in estuarine systems, where a combination of short-term and seasonal drivers influences shoreline position. Tidal anomalies and high-water episodes can shift shoreline position over days to weeks (https://tidesandcurrents.noaa.gov/). Multi-day extratropical storms such as nor’easters generate prolonged wind, surge, and wave energy^9^ that can rapidly reshape low-elevation landforms^10^. Sugarloaf Island, a ~14 ha low-elevation barrier island in Bogue Sound, North Carolina, demonstrates these vulnerabilities (Fig. 1). Located within 100 m of Morehead City, the island lacks dunes and receives minimal sediment input. It has experienced sustained shoreline retreat averaging ~3 m yr−¹, accompanied by marsh and upland habitat loss^11^. These trends underscore the island’s limited capacity for natural recovery and the need for targeted monitoring and intervention.

**Fig. 2:**
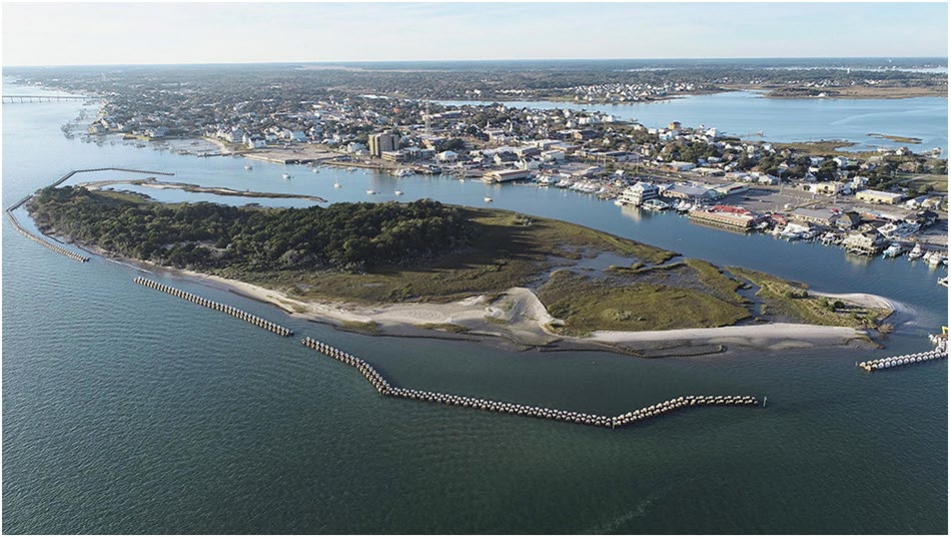
Drone image captured at ~100 m altitude showing Sugarloaf Island, North Carolina, on November 17, 2024 (4 p.m. EST). The image depicts the completion phase of hybrid shoreline stabilization, with Wave Attenuation Devices (WADs) and oyster breakwaters installed along the island’s south, east, and west shorelines; only the north shoreline remains unstructured.

A nature-based shoreline stabilization project was launched on Sugarloaf Island in 2023 to slow erosion and reduce sediment loss. By Winter 2025, the project included ~1000 m of offshore Wave Attenuation Devices (WADs) (https://livingshorelinesolutions.com/; https://seaandshoreline.com/), ~740 m of oyster breakwaters (https://www.sandbaroystercompany.com/), and ~165 m of riprap (Figs. 1d–f, 2). The hybrid nature-based shoreline protection aims to dampen wave energy, trap sediment, and enhance habitat stability while avoiding the ecological tradeoffs of traditional shoreline hardening. Hybrid nature-based shoreline protection leverages the adaptive capacity of ecosystems such as salt marshes and oyster reefs, which stabilize sediment while providing additional habitat benefits^12^. However, the geomorphic performance of hybrid configurations, combining natural and engineered elements, remains under-documented in low-energy estuarine settings^13^ where elevation changes are subtle and seasonally variable.

We conducted 16 high-resolution drone surveys between Summer 2023 and Spring 2025 across east (~3 ha) and west (~1.5 ha) island segments (Fig. 1c), capturing geomorphic and habitat change through pre-, during-, and post-installation phases of hybrid infrastructure. For each interval, we generated orthomosaics, dense point clouds, and DEMs using an SfM-MVS photogrammetric workflow and spatially referenced the data using RTK-GNSS ground control. We implemented rigorous quality control protocols to achieve sub-decimeter horizontal and vertical accuracy.

This dataset offers high-frequency, workflow-aligned observations across eight seasonal intervals. It enables the reliable detection of short-term morphological and ecological responses that are often missed in conventional post-construction assessments. By capturing change continuously over multiple phases, our dataset addresses known monitoring gaps in nature-based shoreline stabilization, where baseline conditions and temporal coverage are often limited^14^. Our monitoring approach aligns with recent recommendations to integrate multi-phase monitoring into shoreline stabilization projects to support adaptive management and long-term success^15^.

All data products are georeferenced to NAD83(2011) UTM Zone 18 N (EPSG:6347), with elevations referenced to NAVD88 using GEOID18A. We prepared and hosted the dataset in a FAIR-compliant repository with complete metadata, structured folder organization, and citation-ready identifiers^16^. It offers a transferable model for high-resolution, seasonally structured drone monitoring of hybrid living shorelines in low-relief coastal environments. The dataset provides quantitative observations of shoreline morphology, habitat extent, and stabilization performance, forming a consistent basis for coastal monitoring and modeling. While the dataset supports ecological applications, these applications are spatial rather than spectral. Because flights prioritized geometric accuracy over radiometric calibration, the imagery is optimized for object-based or morphological analyses of habitat extent and change.

This workflow integrates repeat-survey drone acquisition with consistent altitude, overlap, and sensor parameters; RTK-GNSS control validated against NOAA geodetic guidelines using RMSE and peak-to-peak error metrics; SfM-MVS photogrammetry anchored to a seasonally re-surveyed control network; and Monte Carlo–based georeferencing validation with FAIR-aligned data curation and dissemination. Together, these components provide an uncommon level of temporal consistency, positional accuracy, and methodological transparency for quantitative coastal resilience monitoring. Workflow transferability refers to the reproducibility of methods rather than the generalization of site-specific outcomes. The procedures are best suited for low-energy estuarine or back-barrier environments where stable water clarity, minimal canopy obstruction, and moderate surface roughness support reliable tie-point generation. Parameters such as flight altitude, ground control point quantity and spacing, and overlap ratio can be adjusted to local topography, vegetation, and operational constraints.

## Methods

### Overview and survey design

**Fig. 3:**
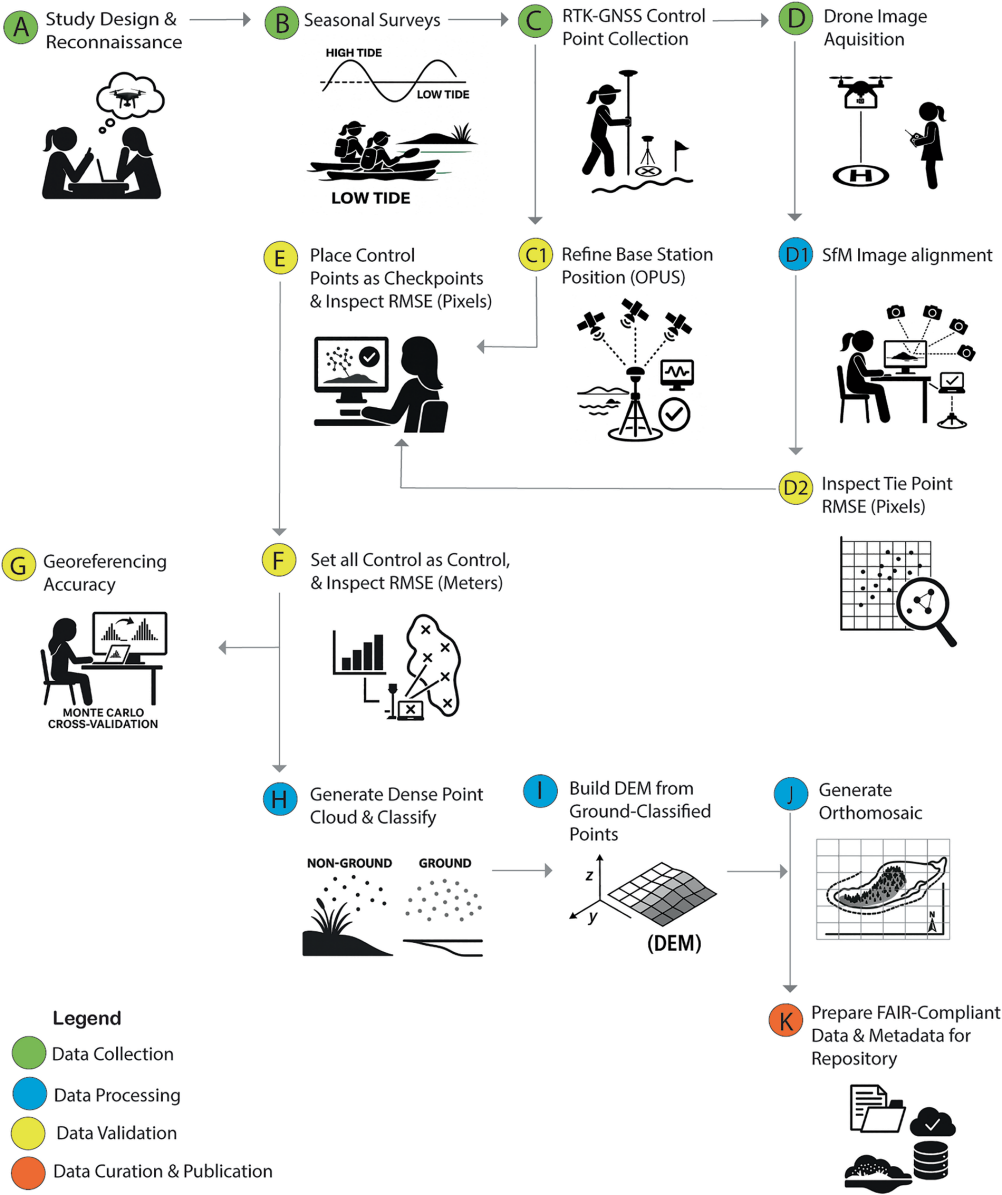
Workflow for generating seasonal datasets using RTK-GNSS and drone-imagery-based SfM-MVS photogrammetry. The workflow spans data collection (green), processing (sky blue), accuracy validation (yellow), product generation (sky blue), and FAIR-aligned data curation (salmon). It was applied to 16 shoreline surveys, 8 targeting the west segment and 8 targeting the east segment of Sugarloaf Island, conducted before, during, and after installation of Wave Attenuation Devices (WADs) and oyster breakwaters as part of a multi-phase hybrid nature-based shoreline stabilization project (2023–2025).

Building on the monitoring framework described above, we developed a workflow, highlighted in Fig. 3, that included data collection, data processing, data validation, and FAIR-aligned data curation. This workflow was used to generate seasonally structured drone data with sub-decimeter resolution and accuracy to document shoreline morphology and intertidal habitat structure at Sugarloaf Island before, during, and after a hybrid living shoreline project.

**Fig. 4:**
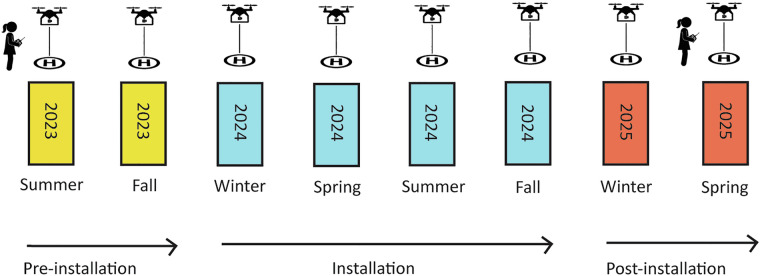
Chronological timeline of seasonal surveys and stabilization phases at Sugarloaf Island, NC. Each survey event (represented by drone takeoff icons) corresponds to paired monitoring of the west and east shorelines. Baseline data were collected in Summer and Fall 2023 (yellow), with post-installation monitoring continuing in Winter and Spring 2025 (salmon).

Illustrated by workflow step A and B in Fig. 3, we conducted 16 drone surveys—eight each on the island’s east and west segments—that capture seasonal monitoring intervals from Summer 2023 through Spring 2025. The surveys were aligned with critical phases of hybrid living shoreline installation and predicted low tides (NOAA Station ID: 8656483, Beaufort, NC). Baseline surveys were conducted in Summer and Fall 2023, with post-stabilization monitoring continuing in Winter and Spring 2025 (Fig. 4). The resulting data products capture the full range of geomorphic and ecological conditions observed on Sugarloaf Island, including bare sediment, vegetated marsh, oyster beds, intertidal flats, shallow subtidal zones, and engineered features such as WADs and oyster breakwaters.

### RTK-GNSS ground control surveys and geolocation framework

Next in workflow steps C and C1 (Fig. 3), we established a high-precision geolocation control framework for remote sensing applications using a Trimble Spectra Precision SP80 RTK-GNSS system, with reported horizontal and vertical RMSEs of 3 mm and 3.5 mm, respectively^17^. Initially, a mainland benchmark was created at a waterfront park (see marked by a yellow star in Fig. 1c-d) to derive a high-accuracy geodetic position from local base station GNSS measurements and Online Positioning User Service (OPUS; https://www.ngs.noaa.gov/OPUS/) processing. However, a network-based correction workflow using the North Carolina Continuously Operating Reference Station system (NC-CORS) was ultimately adopted to eliminate the need for a physical base station. For each NC-CORS-based survey (Summer 2023 east segment to Spring 2025), we surveyed the previously established mainland benchmark and three additional NC-CORS-derived control points to assess the network’s consistency.

Ground Control Points (GCPs) were placed across each island segment (east and west) to capture topographic and surface variability, including elevation extremes, slope breaks, and diverse surface conditions. Each GCP consisted of a 0.25 m × 0.25 m high-visibility orange and black target secured with survey-grade nails. Although the west segment is smaller (1.6 hectares vs 3 hectares), GCP distribution was guided by surface complexity and image coverage, not area alone. The number of GCPs per survey varied by segment and season, ranging from 25 to 51 points.

GCP XYZ coordinates were collected using the Trimble SP80 receiver connected via secure network login credentials to the nearest NC-CORS reference station (“NCBE” in Beaufort, NC; https://geodesy.noaa.gov). In September 2024 “NCBE” was discontinued, requiring a switch to the next closest CORS reference station (“CALO” in Harkers Island, NC or “NCCI” in Cedar Island, NC; https://ncgs.state.nc.us/pages/CORS-and-GNSS.htm) for the Fall 2024 through Spring 2025 surveys. All GCP observations used ~60-epoch averaging and were recorded under high-precision conditions, with positional dilution of precision (PDOP) values below 1.6 and a minimum of 11 satellites used at each fixed location.

### Drone image acquisition

Following workflow step D (Fig. 3), a DJI Phantom 4 Pro drone was used for all image acquisitions due to its proven reliability, portability, and high-resolution RGB sensor suitable for topographic change detection and 3D surface reconstruction. The drone features a 1-inch CMOS RGB sensor with 20 MP resolution, 24 mm equivalent fixed focal length, and a global shutter. Its adjustable aperture (f/2.8–f/11) and ISO range (100–6400) allow it to adapt to variable lighting conditions. Radiometric calibration was not performed because the study prioritized geometric reconstruction and spatial change detection.

All flights were conducted in accordance with Federal Aviation Administration (FAA) Part 107 regulations, with a Visual Observer present to maintain line-of-sight and assist with airspace awareness. Missions were scheduled to coincide with low tide using NOAA tidal predictions (Beaufort Station ID: 8656483) and were conducted under generally stable atmospheric conditions. Wind speeds were limited to <13 kt to minimize motion blur, and overcast conditions were preferred to reduce harsh shadows that interfere with tie point detection during SfM processing. Pre-flight preparations included Notices to Airmen (NOTAMs), METAR/TAF aviation forecasts, and tide tables to ensure compliance and mission safety.

The first eight surveys (Summer 2023–Spring 2024) used DJI Ground Station GS Pro (v2.0) for automated flight planning, allowing pilot control over altitude, speed, overlap, and camera orientation. These flights employed a cross-shore flight pattern, with paths oriented approximately perpendicular to the shoreline, while the camera remained about 20° from nadir to maintain consistent cross shore ground sampling and tie-point geometry. Following the discontinuation of GS Pro, the remaining eight surveys (Summer 2024–Spring 2025) were conducted using Pix4D Capture Pro (v1.4.1), which enforced a parallel flight pattern oriented along primary landscape features and facing the center of the grid^18^. Although the flight orientation changed, gimbal pitch, overlap ratios, flight altitude, and ground sampling distance (GSD) remained consistent across surveys.

All missions were flown at 25 m above ground level to optimize tradeoffs between image coverage, low-tide exposure, and photogrammetric accuracy. This altitude yielded a GSD of approximately 0.0073 m/pixel, calculated automatically within both flight planning platforms based on focal length, sensor dimensions, and resolution. Shutter speed and ISO were set to automatic to accommodate lighting variations, while focus was manually fixed at infinity to maximize image sharpness. Although camera settings and flight parameters were standardized, natural variability in atmospheric conditions, solar angle, and surface wetness introduced minor variation in image texture and photogrammetric tie point density. These sources of variability were addressed during the following quality control and uncertainty analysis procedures.

### Photogrammetric workflow (Structure-from-Motion and Multi-View Stereo)

Drone imagery was processed using a Structure-from-Motion (SfM) and Multi-View Stereo (MVS) photogrammetric workflow to generate high-resolution geospatial datasets. SfM estimates camera parameters and a sparse three-dimensional point cloud through feature detection, image matching, and bundle adjustment^19^. MVS then densifies this sparse reconstruction by calculating depth information from multiple overlapping views to produce the final dense point cloud and digital elevation model^20,21^. This combined SfM–MVS process is commonly referred to as a photogrammetric workflow^7,22^. In this study, image processing was conducted using Agisoft Metashape Professional (v1.6), following standardized photogrammetric workflows^23,24^. The following subsections describe the SfM alignment and MVS densification stages in detail.

### Image alignment and sparse point cloud optimization

In workflow step D1 (Fig. 3), images were first evaluated for quality using Agisoft Metashape’s built-in quality scoring system. Images with quality scores below 0.65 were excluded to minimize distortion and poor feature matching. Alignments were run in ‘Highest Accuracy’ mode with a key point limit of 50,000 and a tie point limit of 5,000 to promote stable image networks and accurate camera positions^25^. A bundle adjustment was performed using a least squares optimization^26^ to refine internal geometry and camera calibration (Fit f, Fit k1, Fit k2, Fit k3, Fit cx, cy, and Adaptive camera model fitting).

Highlighted by workflow step D2 (Fig. 2), sparse point clouds were visually inspected and filtered in Metashape to remove tie points with high reprojection error and unstable geometry, following best practices adapted from^27^. These steps also addressed image texture inconsistencies introduced by variable solar angles and atmospheric conditions, which can affect feature matching and tie point reliability even when flight and camera settings are consistent. Bright sunlight and residual surface wetness from high tides reduced texture contrast on sand-dominated or homogeneous surfaces. These conditions occasionally resulted in sparse or uneven tie point distributions across image sets. We mitigated these effects during the sparse cloud optimization phase by filtering high-error tie points and systematically reviewing alignment diagnostics, including reprojection error histograms and tie point density maps.

Next, in workflow step E (Fig. 3) GCPs were manually identified in the aligned imagery. A free bundle adjustment was performed to calibrate GCP positions within the image network, and pixel error values were used to represent marker accuracy in pixels. Due to the spatial extent of the east segment, image alignment was conducted in two separate Metashape projects. For accurate spatial alignment between these chunks, co-registration was performed using Metashape’s “Align Chunks” function, using shared GCPs and overlapping sparse point features. This step maintained geometric consistency prior to merging the projects into a unified workspace for dense reconstruction. Then in workflow step F (Fig. 2), all GCPs were linked and set as ‘control’ using their RTK-GNSS coordinates to georeference the imagery to NAD83(2011) UTM Zone 18 N (EPSG:6347), with vertical elevations referenced to NAVD88 using GEOID18A. A second bundle adjustment was then conducted with all GCPs incorporated into the model.

Total GCP positional uncertainty in meters was estimated by summing independent GNSS error components in quadrature, including horizontal and vertical rover precision and base station RMSE:

1 $${U}_{{Total}}=\sqrt{{{{{(s}_{1})}^{2}+{(s}_{2})}^{2}+\cdots {(s}_{n})}^{2}}$$

Here *s*_*1*_ represents any individual, independent source of error, such as horizontal or vertical RTK-GNSS precision, OPUS-derived benchmark RMSE, or image measurement uncertainty. *U*_*Total*_ represents total uncertainty. This equation assumes uncorrelated, normally distributed errors^28^ and follows established error propagation principles. Such treatment of positional accuracy is consistent with broader applications of RMSE in coastal vulnerability mapping^29^. Marker and tie point accuracy (in pixels) were defined relative to image network quality, and the same values were applied across all GCPs within each model. These pixel-based accuracy estimates governed how image-based points contributed to internal model geometry, while sub-decimeter-level uncertainty defined the absolute spatial influence of GCPs during georeferencing. The resulting horizontal and vertical GCP uncertainties for each survey are reported in the Technical Validation section, following the completion of the photogrammetric workflow.

### Georeferencing accuracy using monte carlo simulation

To assess georeferencing accuracy (workflow step G, Fig. 3), a Monte Carlo cross-validation approach was implemented using Agisoft Metashape (v1.6), following established methods^7,27^. A Python script adapted from James *et al*.^27^. was used to automate repeated bundle adjustments and RMSE calculations via the Metashape Python API. For each survey, 100 independent bundle adjustment simulations were performed. In each trial, 70% of available GCPs were randomly selected to georeference the model via bundle adjustment, while the remaining 30% were withheld as independent checkpoints. Horizontal and vertical RMSE values were calculated based on residuals between the checkpoint coordinates and model-derived positions.

Final accuracy metrics were computed as the mean RMSE across all 100 simulations for each survey. This ensemble approach reduces sensitivity to specific GCP configurations and avoids overfitting, offering a statistically robust estimate of georeferencing uncertainty compared to single-solution RMSE reporting. Full RMSE distributions are reported in the Technical Validation section.

### Dense point cloud, dem, and orthomosaic generation

After georeferencing, Multi-View Stereo (MVS) was used in Agisoft Metashape to generate dense point clouds for each survey, using “High” quality settings and “Aggressive” depth filtering to retain surface detail while minimizing noise (workflow step H, Fig. 3). Dense reconstruction was performed using the optimized camera parameters derived from the GCP-constrained bundle adjustment. Each survey resulted in a full, unclassified (“raw”) dense point cloud that preserves all surface features and point classifications as initially reconstructed.

Manual cleaning was performed on each dense point cloud to remove outliers and reconstruction artifacts. Ground points were classified using Metashape’s “Classify Ground Points” tool with default parameters (e.g., maximum angle, maximum distance, cell size), which were reviewed and adjusted as needed to account for local surface variability. A separate ground-classified point cloud was produced for each dataset to support digital elevation modeling. DEMs and orthomosaics were subsequently generated using Metashape’s “Build DEM” and “Build Orthomosaic” tools (workflow steps I-J, Fig. 3). Ground-classified points served as the basis for DEM generation and for surface interpolation using the Natural Neighbor method. For DEMs, interpolation was limited strictly to assigning cell values from the nearest ground points without filling large voids or applying additional smoothing. These DEMs, derived directly from ground-classified points, are included in the repository to preserve the original elevation integrity for quantitative analysis.

## Data Records

The complete drone-based shoreline monitoring dataset is archived in Figshare^30–38^ which can also be accessed through the Seasonal Drone Mapping Dataset Collection (10.25452/figshare.plus.c.8101450).

### Survey folders and naming convention

Each survey folder (e.g., *Summer2024*) contains two subfolders corresponding to the east and west shoreline segments. Within each segment, files are organized by data type using a standardized structure: *01_DroneImagery* for raw imagery, *02_RTK_GNSS* for GNSS control inputs (includes point IDs, timestamps, UTM coordinates, NAVD88 elevations, and quality metrics), *03_PointCloud* for full dense point clouds and ground only point clouds, *04_DEM* for digital elevation models, and *05_Orthomosaic* for orthorectified imagery. Two additional folders support data interpretation and reuse: *06_Metadata* contains structured documentation of flight parameters, environmental conditions, RTK-GNSS configuration, and SfM-MVS processing settings, while *07_NBS_Features* stores shapefiles mapping structure placement for that survey date, including WADs, oyster breakwaters, and riprap. To promote transparency and reuse, the root directory includes *00_SOP_DroneCuration*, which contains a standardized curation template, example metadata, and plain-text README files aligned with the SOP.

### Geospatial data products

Each seasonal drone survey produces a consistent suite of geospatial products, including dense and ground-classified point clouds (provided in compressed LAZ format, LAS 1.4, Point Data Format 7), orthomosaics, and DEMs (GeoTIFF format). These data products are derived from RTK-GNSS corrected drone imagery and processed using SfM-MVS photogrammetry and are projected in NAD83(2011)/UTM Zone 18 N (EPSG:6347), with vertical elevations referenced to NAVD88 using the GEOID18A model to maintain spatial consistency across surveys. Each shoreline segment folder also includes a shapefile in *07_NBS_Features* documenting the spatial footprint of structures, WADs, oyster breakwaters, and rip-rap, captured during the monitoring period. These shapefiles were initially digitized from engineering design plans and refined using orthomosaics and field observations to represent as-built placement. This spatial layer enables time-aligned co-analysis of shoreline and habitat change alongside stabilization interventions.

### Metadata files

Each segment folder includes a metadata file (*06_Metadata.docx*) that documents all survey-specific parameters in a structured and standardized format. These metadata files detail the flight platform specifications (e.g., DJI Phantom 4 Pro V2), sensor characteristics (e.g., 20 MP RGB, 1-inch CMOS sensor), flight height, image overlap, ground sampling distance (GSD), and camera settings. They also record RTK-GNSS control configuration, and environmental conditions at the time of survey (e.g., wind speed, cloud cover, tidal level). Processing metadata includes the SfM-MVS software version, bundle adjustment settings, point cloud classification method, number of images retained, and results from image network quality control and georeferencing accuracy assessments. These include Monte Carlo RMSE estimates, ground and raw point cloud densities, and DEM resolution. The metadata structure was adapted and modified from the documentation approach used by^39^ to support transparency and reusability in shoreline monitoring. These metadata files enhance compliance with the FAIR principles^16^ by clearly documenting provenance, acquisition context, and processing decisions in a consistent and transparent format.

## Data Overview

The time series comprises 16 high-resolution drone surveys conducted at Sugarloaf Island, North Carolina, from Summer 2023 to Spring 2025, spanning pre-installation, active construction, and post-installation phases of a hybrid living shoreline project. Data products include dense and ground-classified point clouds, orthomosaics, and digital elevation models generated from RTK-GNSS–controlled SfM-MVS photogrammetric workflow. All data are referenced to NAD83(2011) UTM Zone 18 N and NAVD88 GEOID18A to ensure spatial consistency across the monitoring record. The dataset captures shoreline and habitat change across bare sediment, vegetated marsh, oyster beds, intertidal flats, and engineered shoreline features, providing a seasonally resolved record of geomorphic and ecological responses to shoreline stabilization. Because the RGB imagery was not radiometrically calibrated, pixel brightness values represent relative intensity influenced by illumination and exposure. Users should therefore focus on spatial or geometric analyses, where color assists in visually delineating features but is not treated as quantitative radiance, for example, classifying vegetation, oyster, or sediment zones using texture, tone, or object-based segmentation.

## Technical Validation

### RTK-GNSS accuracy and control network

The RTK-GNSS control network was evaluated across all 16 seasonal surveys using a combined approach of real-time field measurements and benchmark-based validation to ensure both relative precision and absolute positional accuracy. For each survey, ground control points (GCPs) were collected using a Trimble Spectra Precision SP80 GNSS rover connected to the NC-CORS network via the VRS_CMRp protocol. Real-time rover error estimates, recorded during each GCP observation, reflect relative field precision and are reported in Table 1.

**Table 1:** RTK-GNSS accuracy metrics across 16 seasonal surveys, including OPUS-derived benchmark RMSE and peak-to-peak error, as well as real-time rover error estimates for GCP observations.

Survey	Island Segment	Overall RMSE (m)	Peak-to-Peak Errors		Rover Errors		No. of GCPs
			XY RMSE (m)	Z RMSE (m)	XY RMSE (m)	Z RMSE (m)	
2023-08-29	West	0.016	0.003	0.049	0.006	0.007	33
2023-09-12	East	0.017	0.011	0.061	0.008	0.008	49
2023-10-25	West	0.016	0.004	0.067	0.004	0.005	35
2023-10-24	East	0.014	0.000	0.054	0.005	0.006	46
2024-02-20	West	0.013	0.008	0.063	0.007	0.010	25*
2024-02-18	East	0.013	0.004	0.062	0.008	0.012	41
2024-05-23	West	0.015	0.006	0.057	0.007	0.009	44
2024-05-22	East	0.014	0.010	0.068	0.007	0.010	42
2024-09-29	West	0.016	0.012	0.055	0.009	0.012	32
2024-09-28	East	0.014	0.009	0.059	0.012	0.017	45
2024-11-27	West	0.012	0.003	0.053	0.009	0.010	43
2024-11-17	East	0.012	0.001	0.056	0.008	0.011	50
2025-02-24	West	0.013	0.004	0.056	0.007	0.009	47
2025-02-28	East	0.014	0.008	0.056	0.006	0.009	54
2025-05-24	West	0.016	0.003	0.053	0.007	0.009	46
2025-05-07	East	0.014	0.005	0.054	0.008	0.01	49

All values meet NOAA (2024) geodetic survey thresholds.

To assess absolute accuracy, the benchmark defining each survey’s control network was submitted to the Online Positioning User Service (OPUS) for a post-processed geodetic solution. To maintain vertical consistency between rover and benchmark data, a fixed antenna height of 2 m was enforced in all OPUS submissions (matching the rover setup), unless otherwise noted in the site log. Benchmark accuracy was further validated using the User-Friendly CORS (UFCORS) utility, which estimates coordinate accuracy based on the three nearest NC-CORS reference stations. Standardized metadata, including start time, observation duration, reference station ID, and sampling rate, were included with each submission.

Two complementary metrics were used to evaluate benchmark accuracy: 1) overall root mean square error (RMSE), calculated as the root-mean-square difference between GNSS observations and known benchmark coordinates across all logged epochs, and 2) peak-to-peak error, defined as the range of observed values during each OPUS session and computed independently for the three surrounding NC-CORS baselines^40^. Benchmark-derived values express the absolute spatial reliability of the control network, while real-time rover error estimates reflect the relative precision of each GCP observation.

Following NOAA’s guidelines for geodetic-quality surveys, we adopted thresholds of an overall RMSE ≤0.03 m, horizontal (XY) peak-to-peak error ≤0.04 m, and vertical (Z) peak-to-peak error ≤0.08 m^41^. As shown in Table 1, all surveys met these criteria: overall RMSE values were consistently low (<0.017 m), and vertical peak-to-peak errors ranged between 0.049 and 0.068 m across all seasonal campaigns. This consistency across all survey periods supports the reliability of the control strategy and its use in high-confidence shoreline change detection.

### Repeated control point validation of NC-CORS network

To independently assess the positional repeatability of the NC-CORS correction system across seasonal surveys, we evaluated 39 repeated RTK-GNSS occupations of fixed control points between Fall 2023 and Spring 2025. These observations span multiple NC-CORS reference stations, including NCBE and CALO, with NCCI used during late 2024–2025. All surveys were conducted using a Trimble SP80 receiver connected via the VRS_CMRp mount point, with real-time corrections applied during acquisition. Observations were collected under favorable satellite geometry (PDOP < 1.6; ≥11 satellites) using ~60-epoch averaging per point to reduce multipath and short-term noise.

For each control point, horizontal (ΔXY) and vertical (ΔZ) deviations were computed by subtracting seasonal coordinates from that point’s multi-survey mean position. Horizontal deviation was calculated as the Euclidean distance from the mean in X and Y (i.e., root-sum-square). Each observation was classified into one of three overall quality tiers based on its combined positional error across all three dimensions (XYZ), using unrounded deviation values. Thresholds were defined as: High precision (≤0.03 m in both horizontal and vertical components), Moderate precision (0.03–0.05 m), and Flagged (>0.05 m). These classifications reflect total positional quality and are summarized in Table 2.

**Table 2:** Summary statistics for each control point surveyed with NC-CORS RTK GNSS from 2023–2025.

Control Point	n	Mean Z (m)	Std Dev Z (m)	Max ΔZ (m)	Mean ΔXY (m)	Max ΔXY (m)	% XYZ High	% XYZ Moderate	% XYZ Flagged
BASE	9	1.4406	0.0183	0.0375	0.0213	0.0617	78	11	11
CP1	12	1.4769	0.0304	0.0589	0.0220	0.0585	50	33	16
CP2	6	1.5551	0.0183	0.0244	0.0341	0.0577	50	33	16
CP3	12	1.4752	0.0278	0.0474	0.0248	0.0579	58	25	16

Z repeatability is expressed as standard deviation and max vertical deviation from mean (|ΔZ|). ΔXY denotes Euclidean distance from each control point’s mean UTM position. Quality classifications were based on thresholds applied to unrounded deviation values: High (≤0.0300 m in both ΔXY and |ΔZ|), Moderate (between 0.0300 and 0.0500 m), and Flagged (>0.0500 m).

**Fig. 5:**
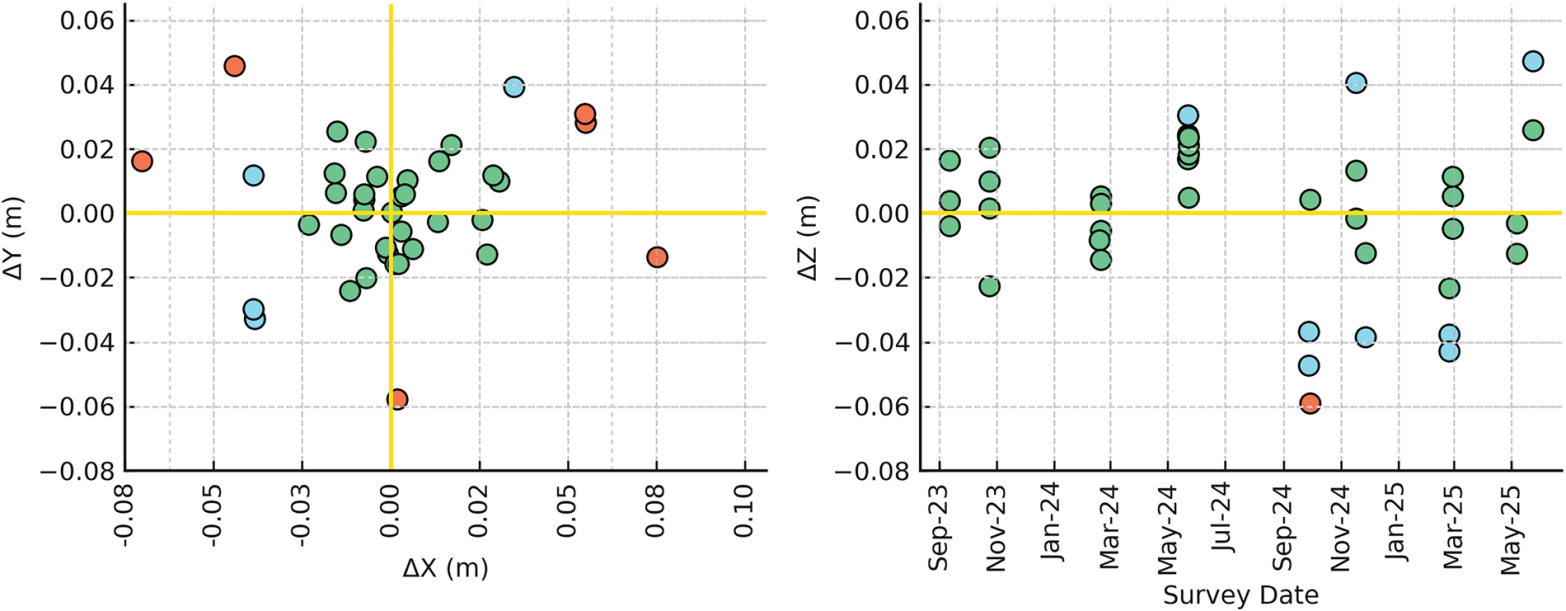
Horizontal and vertical repeatability of NC-CORS-based RTK-GNSS control points across 15 seasonal surveys from 2023 to 2025. The left panel shows planimetric error (ΔX vs. ΔY) relative to each control point’s mean position, and the right panel shows vertical deviation (ΔZ) over time. Each observation is color-coded by positional accuracy: sea green indicates high-precision results (≤0.03 m), sky blue indicates moderate precision (0.03–0.05 m), and salmon indicates flagged observations (>0.05 m), with classifications applied independently to horizontal and vertical components.

**Fig. 6:**
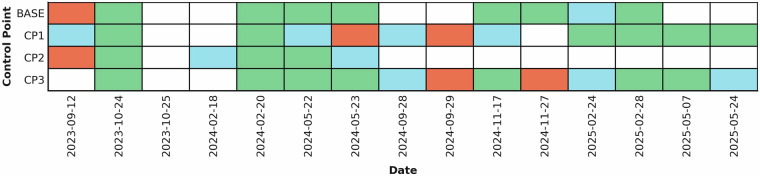
Heatmap showing the per-survey positional quality of GNSS observations collected at four fixed control points over 15 campaigns (2023–2025). Each colored cell represents a GNSS measurement for a specific control point on a particular date, with colors indicating horizontal and vertical accuracy: sea green (≤0.03 m), sky blue (0.03–0.05 m), and salmon (>0.05 m). White cells indicate dates with no accepted observation for that control point—either the point was not occupied, or an observation was collected but failed quality control and was removed (e.g., disturbance from construction/multipath).

The geodetic performance of the NC-CORS control framework was consistent with published RTK-GNSS benchmarks, which report horizontal repeatability of 0.01–0.02 m and vertical deviations of 0.03–0.04 m^42,43^. Across all 39 occupations, 76% of horizontal positions and 83% of vertical positions met the high-precision threshold (≤0.03 m), with flagged observations occurring in fewer than 7% of cases (Fig. 5). These flagged points were typically associated with semi-obstructed control points, such as CP3 in September 2023, where local construction and nearby buildings likely introduced multipath effects. Notably, these outliers were not accompanied by poor PDOP values or satellite availability, suggesting site-specific interference rather than deficiencies in the correction service. In addition, the consistent recurrence of high-precision observations across campaigns and control points reinforces the spatial reliability of the NC-CORS correction for long-term multi-temporal remote sensing applications (illustrated as sea green in Fig. 6), which has also been employed in previous coastal drone monitoring studies^44^.

### Drone photogrammetric processing assessment

Drone-based SfM accuracy was evaluated using internal diagnostics and ground-truth control. The following metrics were assessed: (1) reprojection error, (2) GCP marker accuracy within the image network, and (3) RTK-GNSS-derived ground error for each control point. For each survey segment, we report the reprojection RMSE and GCP image RMSE (both in pixels), along with the GNSS-derived GCP ground RMSE (m) and total number of GCPs used in the SfM reconstruction (Table 3).

**Table 3:** Photogrammetric quality metrics across all drone surveys.

Survey	Island Segment	Image coordinates (pix)		GCP ground RMSE (m, XY/Z)	No. of GCPs GCPs
		Reprojection RMSE	GCP image RMSE		
2023-08-29	West	0.548	0.291	0.017/0.018	33
2023-09-12	East	0.442	0.271	0.018/0.018	49
2023-10-25	West	0.484	0.300	0.017/0.017	35
2023-10-24	East	0.434	0.253	0.015/0.015	46
2024-02-20	West	0.508	0.325	0.015/0.016	25
2024-02-18	East	0.494	0.231	0.015/0.017	41
2024-05-23	West	0.479	0.263	0.017/0.018	44
2024-05-22	East	0.494	0.260	0.016/0.017	42
2024-09-29	West	0.511	0.213	0.018/0.020	32
2024-09-28	East	0.453	0.199	0.017/0.018	43
2024-11-27	West	0.681	0.202	0.015/0.016	43
2024-11-17	East	0.473	0.220	0.015/0.016	51
2025-02-24	West	0.435	0.246	0.015/0.016	47
2025-02-28	East	0.489	0.310	0.015/0.017	54
2025-05-24	West	0.416	0.189	0.017/0.018	46
2025-05-07	East	0.505	0.216	0.017/0.018	49

Values represent reprojection and GCP image error in pixels, ground-based GCP RMSE in meters (horizontal/vertical), and total number of GCPs used per segment. Ground RMSE values are computed using Eq. 1 as the quadrature sum of horizontal and vertical error components per survey.

Reprojection error, calculated within Agisoft Metashape (v1.6), represents the distance (in pixels) between a reconstructed 3D point projected onto an image and its originally observed location. This metric quantifies the internal consistency of the photogrammetric model and is sensitive to image texture, camera calibration, and tie-point distribution. Reprojection errors <1 pixel are generally indicative of good alignment and robust bundle adjustment^25^. Across the 16 segment-level models processed in this study, reprojection RMSE values ranged from 0.416 to 0.681 pixels (Table 3). All models remained below the 1-pixel threshold, indicating consistently well-converged image networks and acceptable internal geometry across variable lighting and surface conditions.

Marker accuracy, or GCP image error, was also evaluated in pixels and reflects the residual offset between GCPs manually marked in the imagery and their calculated locations in the bundle adjustment solution. These values are directly influenced by image quality, GCP visibility, and calibration error, and were assessed following previous SfM validation efforts^27^ The GCP marker error (image RMSE) remained below 0.325 pixels across all surveys (Table 3), further supporting the accuracy of camera optimization and the integrity of the image network.

In addition to internal image-based metrics, absolute positional accuracy was quantified using RTK-GNSS-derived horizontal and vertical ground errors for each GCP. RTK-GNSS precision was calculated using base station OPUS reports and rover precision estimates, following the same quadrature-based uncertainty propagation approach outlined in the Photogrammetric Workflow section. RTK-GNSS-based GCP ground error (reported in meters) was expressed as a slash-delimited horizontal/vertical RMSE pair for each survey (e.g., 0.016/0.017 m; see Table 3). As noted earlier, all RTK-GNSS observations utilized fixed-integer solutions with a PDOP of ≤1.6 and a minimum of 11 satellites. Together, these metrics confirm strong internal model alignment, georeferencing, and suitability of the products for change detection applications.

### Georeferencing error evaluation

Georeferencing uncertainty was quantified using 100-run Monte Carlo simulations for each survey. In each run, 70% of GCPs were randomly selected to georeference the model, while the remaining 30% were withheld as independent checkpoints. RMSE values were computed for horizontal (XY), vertical (Z), and combined spatial (XYZ) positioning, providing a robust measure of geolocation precision under varying survey conditions (see Photogrammetric Workflow section).

**Fig. 7:**
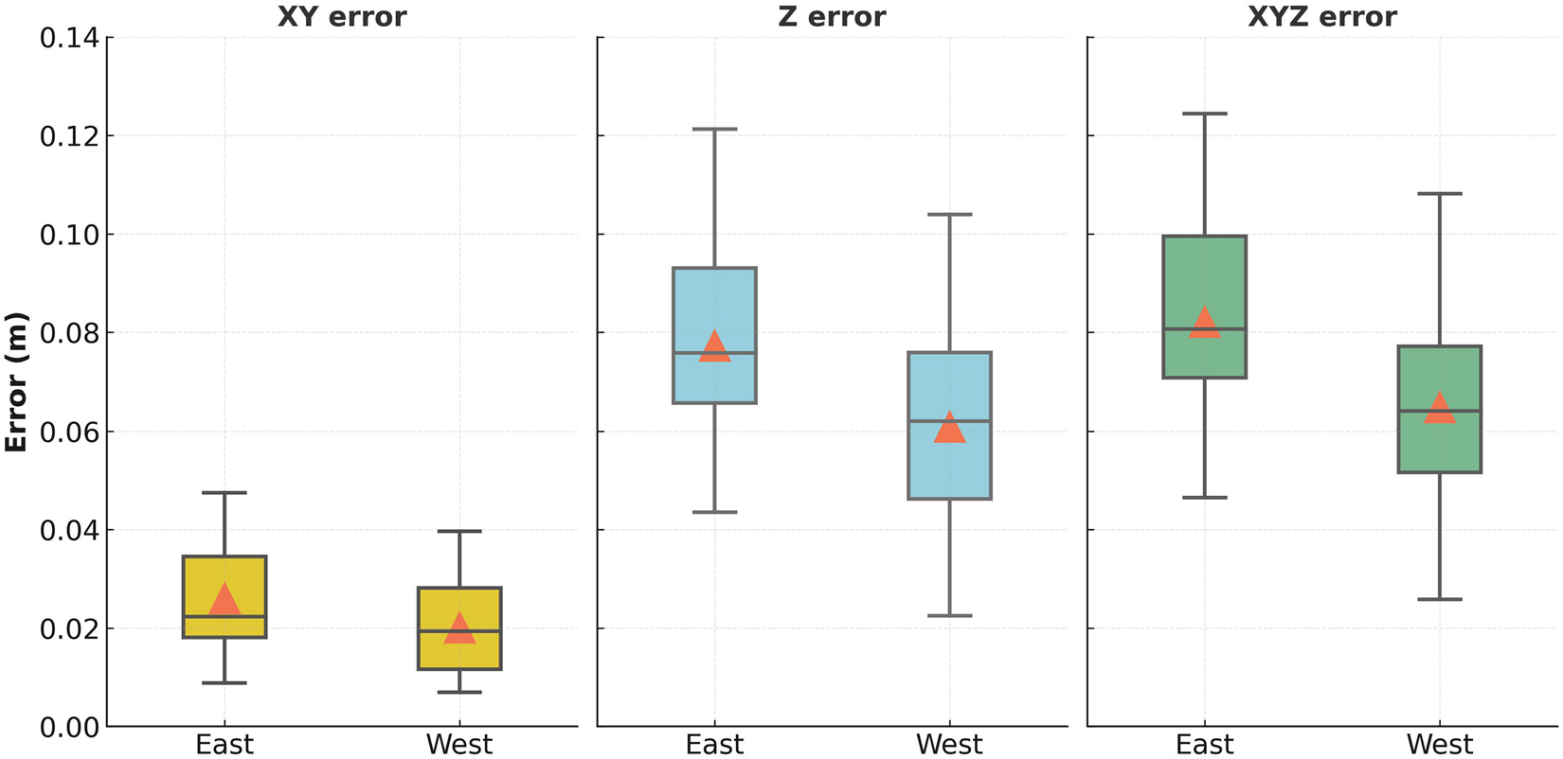
Boxplots of horizontal (XY), vertical (Z), and total spatial (XYZ) RMSE values from 100-run Monte Carlo simulations, grouped by island segment. Each box represents the distribution of RMSEs across all surveys (n = 800 per segment). Orange triangles denote pooled mean values. The west consistently showed lower RMSEs, particularly in the vertical dimension.

Monte Carlo horizontal RMSE values ranged from 0.008 m to 0.044 m, and vertical RMSE values from 0.030 m to 0.089 m, with most standard deviations below 0.005 m, indicating high consistency across simulations (Table 4). The lowest horizontal error occurred in the west segment in May 2024 (0.008 m), while the highest vertical error was recorded in Fall 2024 (up to 0.089 m). Most vertical RMSE values clustered between 0.03–0.07 m. These results closely match those of Elsner *et al*.^8^, who reported vertical RMSEs of 0.054–0.113 m using drone–SfM–MVS photogrammetric workflows with high-accuracy GNSS control, further validating our georeferencing workflow. RMSE distributions grouped by island segment are shown in Fig. 7, where the west segment generally exhibited lower RMSEs, with the most consistent differences observed in the vertical dimension.

**Table 4:** Georeferencing uncertainty statistics derived from 100-run Monte Carlo simulations for each drone survey.

Survey	Island Segment	XY Mean (m)	XY SD (m)	XY Median (m)	XY Min (m)	XY Max (m)	Z Mean (m)	Z SD (m)	Z Median (m)	Z Min (m)	Z Max (m)
2023-08-29	West	0.033	0.004	0.034	0.017	0.039	0.053	0.001	0.052	0.049	0.058
2023-09-12	East	0.044	0.002	0.044	0.038	0.047	0.07	0.004	0.072	0.06	0.076
2023-10-25	West	0.011	0.001	0.011	0.008	0.012	0.07	0.002	0.07	0.065	0.078
2023-10-24	East	0.021	0.001	0.021	0.017	0.024	0.076	0.002	0.076	0.069	0.081
2024-02-20	West	0.012	0.001	0.013	0.008	0.014	0.032	0.001	0.032	0.022	0.035
2024-02-18	East	0.017	0.002	0.017	0.011	0.02	0.047	0.002	0.047	0.044	0.051
2024-05-23	West	0.008	0.001	0.008	0.007	0.008	0.062	0.001	0.062	0.054	0.068
2024-05-22	East	0.014	0.001	0.014	0.009	0.015	0.064	0.002	0.064	0.059	0.068
2024-09-29	West	0.026	0.002	0.026	0.02	0.03	0.089	0.008	0.09	0.073	0.104
2024-09-28	East	0.027	0.002	0.027	0.021	0.031	0.089	0.008	0.09	0.073	0.104
2024-11-27	West	0.02	0.001	0.02	0.017	0.022	0.03	0.001	0.03	0.025	0.034
2024-11-17	East	0.02	0.001	0.02	0.017	0.023	0.03	0.002	0.031	0.025	0.034
2025-02-24	West	0.018	0.001	0.018	0.016	0.021	0.061	0.002	0.061	0.055	0.067
2025-02-28	East	0.019	0.001	0.019	0.016	0.021	0.061	0.002	0.061	0.055	0.068
2025-05-24	West	0.031	0.002	0.031	0.024	0.035	0.088	0.005	0.088	0.071	0.097
2025-05-07	East	0.032	0.003	0.032	0.025	0.036	0.088	0.006	0.089	0.072	0.097

Horizontal (XY) error was computed as the root-sum-square of X and Y components. Reported metrics include the mean, standard deviation (SD), median, minimum (min), and maximum (max) error for both horizontal (XY) and vertical (Z) dimensions. Results are shown separately for east and west Island segments to support uncertainty assessment across surveys conducted between Summer 2023 and May 2025.

To evaluate whether environmental variability or the transition between flight-planning software influenced georeferencing precision, metadata-based environmental parameters (tides, wind speed, illumination, tidal stage, and humidity) were extracted for all 16 surveys and compared with corresponding Monte Carlo RMSE values. Flights were conducted under low-to-moderate wind speeds (<13 kt), roughly uniformly illuminated conditions, and near-low-tide exposures (predicted MLLW range = −0.12 to 0.55 m). The transition from DJI GS Pro to Pix4D Capture Pro in Spring 2024 altered flight-path programming but maintained identical altitude, overlap, and image geometry. Seasonal averages (Table 5) show that mean wind ranged from 6 to 13 kt and humidity from 40% to 68%, yet georeferencing accuracy remained consistent across all surveys (mean XY RMSE = 0.011–0.038 m; mean Z RMSE = 0.03–0.089 m). No systematic change in error magnitude or variability was observed following the software transition. These results demonstrate that the photogrammetric workflow maintained sub-decimeter reproducibility under a broad range of environmental conditions and between independent flight-planning platforms.

**Table 5:** Seasonal mean (±1 standard deviation (SD)) environmental conditions and georeferencing accuracy for sUAS-SfM surveys conducted from 2023 to 2025 (*n* = 2 per season).

Season	Control Software	Mean MLLW (m)	Mean Wind (kt)	Mean Humidity (%)	Mean XY RMSE (m)	Mean Z RMSE (m)
Summer 2023	DJI GS Pro	0.127 ± 0.018	8.0 ± 2.83	68.5 ± 2.1	0.038 ± 0.008	0.062 ± 0.012
Fall 2023	DJI GS Pro	0.171 ± 0.044	10.0 ± 0.00	68.0 ± 2.8	0.016 ± 0.007	0.073 ± 0.004
Winter 2024	DJI GS Pro	0.307 ± 0.068	13.0 ± 0.71	58.0 ± 14.1	0.014 ± 0.004	0.040 ± 0.011
Spring 2024	DJI GS Pro	0.223 ± 0.045	7.5 ± 3.54	66.0 ± 0.0	0.011 ± 0.004	0.063 ± 0.001
Summer 2024	Pix4D Capture Pro	0.210 ± 0.00	6.25 ± 1.06	68.5 ± 2.1	0.026 ± 0.001	0.089 ± 0.000
Fall 2024	Pix4D Capture Pro	0.246 ± 0.072	10.25 ± 1.06	54.5 ± 13.4	0.020 ± 0.000	0.030 ± 0.000
Winter 2025	Pix4D Capture Pro	0.061 ± 0.164	8.25 ± 4.60	44.0 ± 24.0	0.018 ± 0.001	0.061 ± 0.000
Spring 2025	Pix4D Capture Pro	0.119 ± 0.038	6.75 ± 1.06	40.5 ± 7.8	0.032 ± 0.001	0.088 ± 0.000

MLLW = Mean Lower Low Water tidal datum. Values represent the mean ± sample standard deviation of wind speed, relative humidity, and Monte Carlo-derived horizontal (XY) and vertical (Z) RMSE. DJI GS Pro was used for surveys through Spring 2024; Pix4D Capture Pro was adopted thereafter.

## Data Availability

The datasets generated and described in this Data Descriptor are archived in Figshare as a public Collection, *Seasonal Drone Mapping Dataset – Sugarloaf Island, NC (2023–2025)*, available at 10.25452/figshare.plus.c.8101450. The Collection contains eight seasonal surveys and their accompanying metadata, as described in the Data Records section.

## Code availability

All analyses used standard, widely available software. Geospatial preprocessing and table formatting were completed using Python tools within ArcGIS Pro (v3.4). Structure-from-Motion georeferencing accuracy was evaluated through a Monte Carlo cross-validation routine implemented with the Agisoft Metashape (v1.6) Python API, based on James *et al*.^27^. The original *Monte_Carlo_BA.py* script by M.R. James was minimally updated for compatibility with Metashape v1.6 (accommodating the module rename from *PhotoScan* to *Metashape*). The updated script, Monte_Carlo_BA_Metashape1_6.py, together with a brief README, is openly available in the repository.
